# Accuracy of joint line restoration based on three-dimensional registration of the contralateral tibial tuberosity and the fibular tip

**DOI:** 10.1186/s40634-021-00400-8

**Published:** 2021-09-29

**Authors:** Sandro Hodel, Anna-Katharina Calek, Philipp Fürnstahl, Sandro F. Fucentese, Lazaros Vlachopoulos

**Affiliations:** 1 Department of Orthopedics, Balgrist University Hospital, University of Zurich, Forchstrasse 340, 8008 Zurich, Switzerland; 2 Research in Orthopedic Computer Science (ROCS), Balgrist University Hospital, University of Zurich, Forchstrasse 340, 8008 Zürich, Switzerland

**Keywords:** Total knee arthroplasty, TKA, Revision TKA, Joint line, Tibial tuberosity, Fibular tip

## Abstract

**Purpose:**

To assess a novel method of three-dimensional (3D) joint line (JL) restoration based on the contralateral tibia and fibula.

**Methods:**

3D triangular surface models were generated from computed tomographic data of 96 paired lower legs (48 cadavers) without signs of pathology. Three segments of the tibia and fibula, excluding the tibia plateau, were defined (tibia, fibula, tibial tuberosity (TT) and fibular tip). A surface registration algorithm was used to superimpose the mirrored contralateral model onto the original model. JL approximation and absolute mean errors for each segment registration were measured and its relationship to gender, height, weight and tibia and fibula length side-to-side differences analyzed. Fibular tip to JL distance was measured and analyzed.

**Results:**

Mean JL approximation did not yield significant differences among the three segments. Mean absolute JL error was highest for the tibia 1.4 ± 1.4 mm (range: 0 to 6.0 mm) and decreased for the fibula 0.8 ± 1.0 mm (range: 0 to 3.7 mm) and for TT and fibular tip segment 0.7 ± 0.6 (range: 0 to 2.4 mm) (*p* = 0.03). Mean absolute JL error of the TT and fibular tip segment was independent of gender, height, weight and tibia and fibula length side-to-side differences. Mean fibular tip to JL distance was 11.9 ± 3.4 mm (range: 3.4 to 22.1 mm) with a mean absolute side-to-side difference of 1.6 ± 1.1 mm (range: 0 to 5.3 mm).

**Conclusion:**

3D registration of the contralateral tibia and fibula reliably approximated the original JL. The registration of, TT and fibular tip, as robust anatomical landmarks, improved the accuracy of JL restoration independent of tibia and fibula length side-to-side differences.

**Level of evidence:**

IV

## Introduction

Restoration of the original joint line (JL) in total knee arthroplasty (TKA) remains crucial for optimal functional and clinical outcome [6] but challenging, especially in revision cases with extensive osseous destruction [24]. Altering the JL effects tibiofemoral kinematics and joint stability adversely [3, 12]. Additionally, a relative lowering of the patella in relation to the JL (pseudo patella baja) can cause anterior knee pain following TKA [2, 9, 10].

The aim is therefore, to restore the original anatomical JL as accurately as possible. For this purpose, various radiological landmarks based on the femur (medial and lateral epicondyle, adductor tubercle) [8, 15] or the tibia and fibula [7] have been described. Limitations of previously described methods are their dependency on on patient height [13, 18]. Moreover, landmarks as the epicondyles are prone to bony destruction and are not always identifiable in a revision setting. To overcome these limitations, Maderbacher et al. proposed to restore the JL based from the contralateral fibular tip using weight-bearing x-rays [14]. Recently, a growing interest has evolved regarding three-dimensional (3D) registration and planning based on the contralateral unaffected anatomy [4, 17]. This method could potentially improve accuracy in JL restoration and be of high interest for planning revision TKA [22]. However, no JL restoration method based on the contralateral 3D registration exists to date, to the best of our knowledge. Moreover, it remains unclear which anatomical landmarks most reliably approximate the JL. Therefore, we hypothesized that the JL can be restored accurately from the contralateral 3D registration including the tibial tuberosity and fibular tip as anatomical landmarks. The aim of the study was to analyze the accuracy of a 3D registration algorithm of the contralateral side to restore the JL using different segments of the tibia and fibula for the registration. Additionally, we investigated the effect of anatomical side-to side differences and patient demographics on the accuracy of the JL restoration.

## Methods

### Specimens and 3D registration algorithm

Ninety-six cadaver specimens of the lower leg, provided by the Institute of Forensic Medicine, University Zurich and analyzed in a previous study [17], were included without previous trauma, surgery or deformity of the tibia or fibula. Thirty-four male and 12 female donors (missing gender information in two specimens) with an average age of 52 years ±17.7 (range: 21 to 95 years) were included. The average weight was 83.1 ± 16.5 kg (range: 55 to 111 kg) and the average height was 176.2 ± 8.6 cm (range: 154 to 195 cm).

**Fig. 1:**
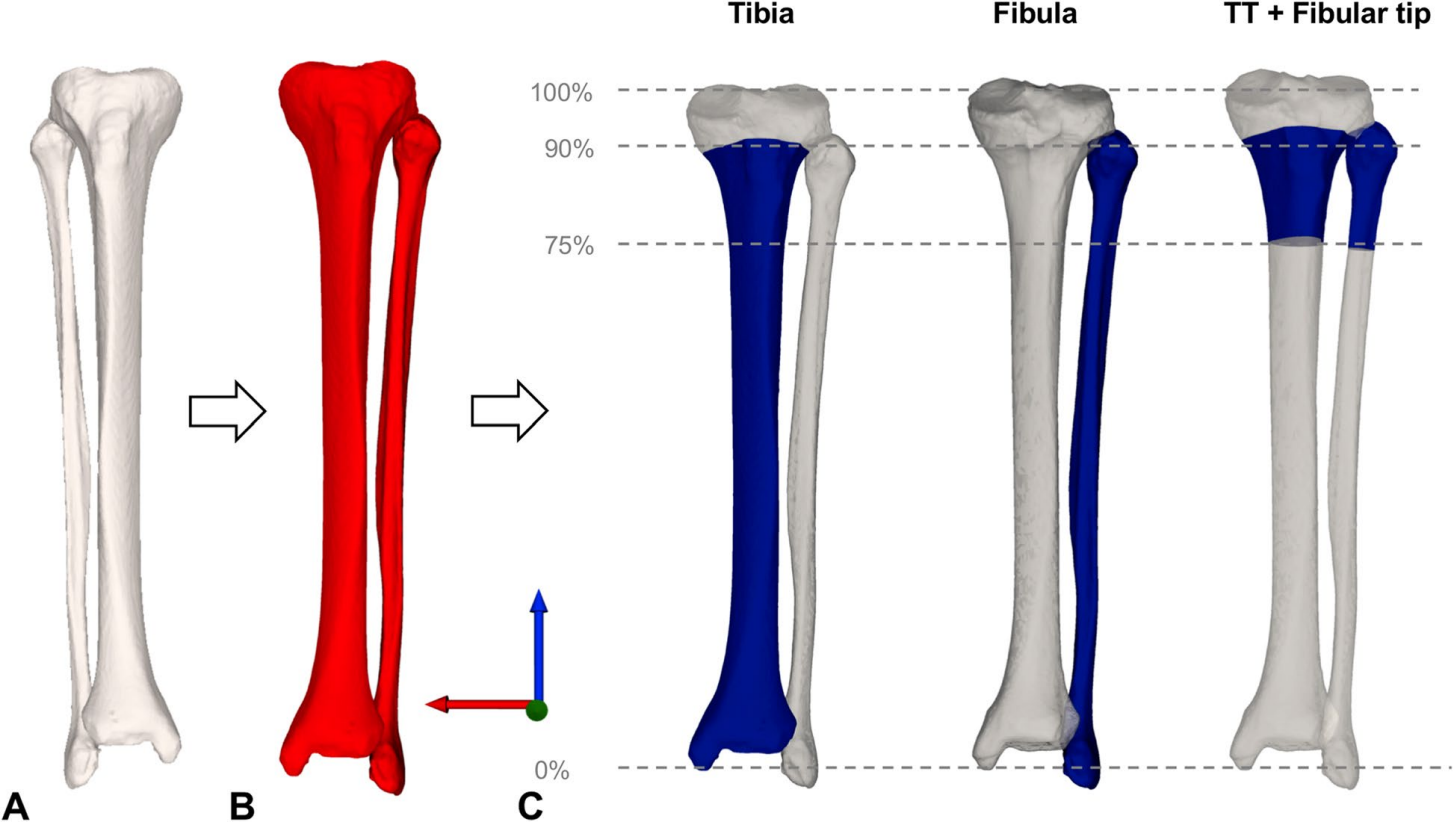
Definition of tibia and fibula segments for contralateral registration. TT: Tibial tuberosity. The contralateral model (white) (**A**) was mirrored (red) (**B**) and three anatomical segments (blue) (**C**) were defined for registration and depicted from left to right: Tibia: including 90% of the tibia length, Fibula: complete fibula model, TT and fibular tip: including the anatomical structures of the TT and proximal fibular tip

High-resolution computer tomography (CT) data were acquired using a Somatom Definition Flash CT scanner (Siemens®, Erlangen, Germany) with a slice thickness ranging from 0.5 to 0.6 mm. 3D triangular surface models of 96 paired (48 left, 48 right) healthy tibiae and fibulae were generated with manual threshold segmentation and region growing using MIMICS software (MIMICS Medical, Materialise NV, Leuven, Belgium) and imported into the in-house surgical planning software CASPA (Balgrist, Zurich, Switzerland). To approximate the original JL from the mirrored contralateral side, an iterative closest point (ICP) algorithm [1] was used to superimpose the mirrored contralateral model onto the original model, as described in previous studies [17, 25]. A 3D coordinate system was defined according to [5]; z-axis equal directional vector as the anatomical tibia axis defined by an oriented bounding box (OBB) [26], x-axis: lateral, y-axis: anterior (see Fig. 1).

### Definition of tibia and fibula segments for contralateral registration

As segment selection and included anatomical structures potentially improve the accuracy to approximate the original model [25], we defined three distinct segments of the lower leg to restore the JL, excluding the potentially deformed tibia plateau. The contralateral lower leg model was mirrored and three anatomical segments were defined (Fig. 1). We included previously described anatomical landmarks as the tibial tuberosity (TT) and fibular tip [18]:

- *Tibia:* The segment was defined as 90% of the tibia length.
- *Fibula:* The segment included the complete fibula model.
- *Tibial tuberosity (TT) and fibular tip: The* segment was defined from 75% to 90% of the tibia length and the complete corresponding proximal fibula segment (see Fig. 1).

The surface registration algorithm to superimpose the mirrored contralateral models onto the original model was repeated for all three defined segments of the tibia and fibula of prespecified lengths, as described above.

### Definition of joint line and accuracy of joint line restoration

**Fig. 2:**
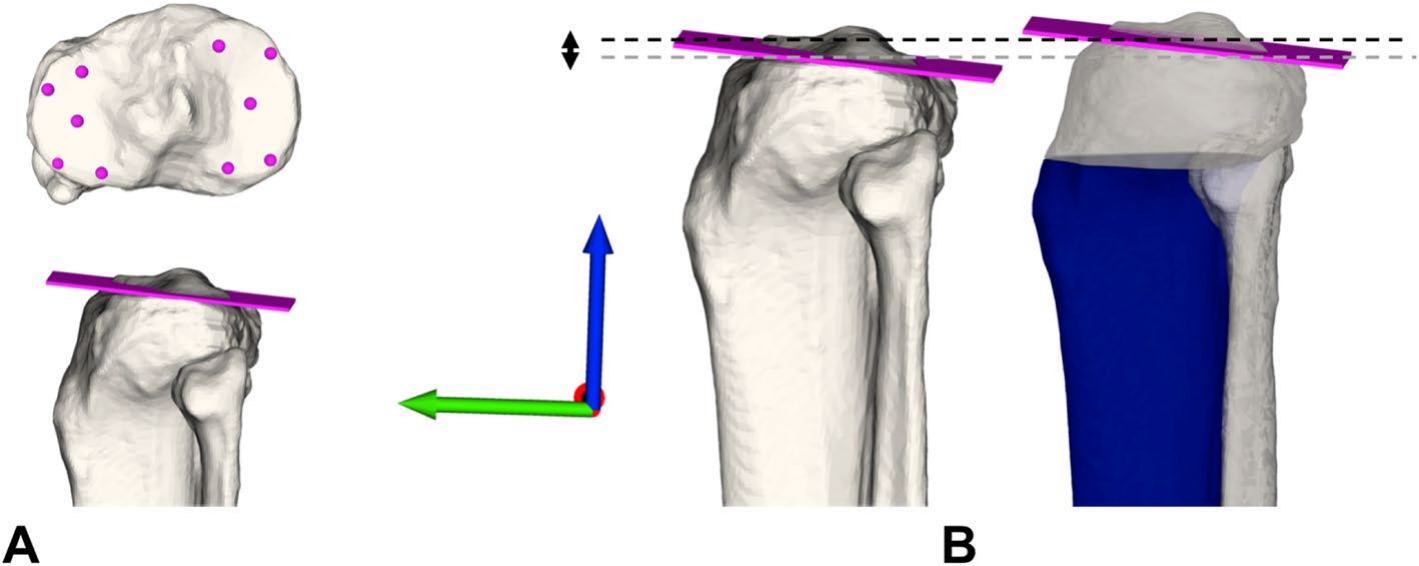
Definition of joint line and accuracy of joint line restoration. **A** Definition of the tibia joint line as the average plane of ten spheres at the surface of the medial and lateral tibial plateau (four at the rim and one at the center each). **B** Tibia JL restoration accuracy measured from original JL left (pink, grey dotted line) after superimposition of the contralateral model (blue, black dotted line) in direction of anatomical tibia axis (blue arrow) in mm (positive values indicating an elevation of the JL, negative values indicating a distalisation)

The JL was defined as the average plane of ten surface registration points on the medial and lateral tibial plateau in a standardized fashion on the rim and the center of the tibial plateau and visualized in Fig. 2. The approximation of the JL from the contralateral side compared to the original JL was measured in mm in direction of the anatomical tibia axis (z-axis) (positive values indicating an elevation of the JL, negative values indicating a distalization) (see Fig. 2B). Additionally, JL error was defined as mean absolute error for each segment.

### Measurement of tibia, fibula length and distance of the fibular tip to the joint line

**Fig. 3:**
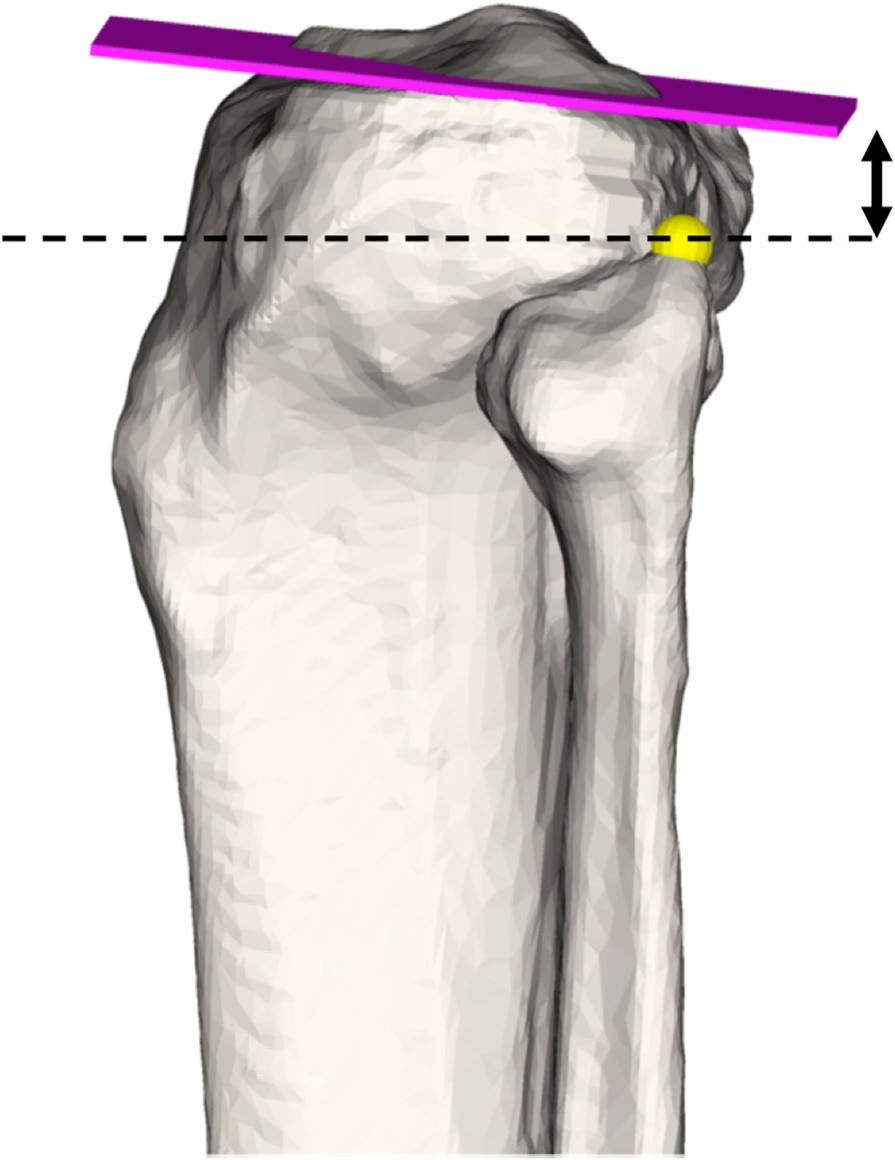
Measurement of fibular tip to joint line distance. Measurement of shortest distance of fibular tip (yellow sphere) to JL (pink) is depicted (black arrow)

The length of the tibia and fibula model was defined by the OBB [17]. Side-to-side differences are reported as mean absolute differences. The closest distance of the fibular tip to the JL was measured using an automatic surface registration sphere on the highest point of the fibular tip (see Fig. 3).

The JL definition and distance of the fibular tip to the JL measurement were performed by two readers in 20 lower legs to assess accuracy and inter-reader reliability. Intra-reader reliability was not performed due to the highly standardized definition of the surfaces and the mostly automatized measurement procedure.

### Statistics

A post-hoc sample size calculation was performed (significance level set: α = 0.05, power level: β = 0.80) to detect a mean JL error of 0.5 mm, assuming a standard deviation of 1 mm. This resulted in a sample size of 36 per group.

Inter-reader reliability was performed using intraclass correlation coefficient (ICC) with a two-way mixed-effect model assuming a single measurement and absolute agreement.

Normal distribution of the data was tested with Shapiro-Wilk’s test and histograms. Data are reported as mean ± standard deviation and range. One-way ANOVA was performed to analyze differences of tibia JL approximations and Kruskal-Wallis for JL error among the three segments. Multiple post-hoc testing was Bonferroni corrected. Differences between gender were analyzed using a non-paired t-test. Gender, height, weight and side-to-side differences of the tibia length, fibula length and fibular tip to JL distance were included in a linear regression model to analyze their influence on JL error and reported as regression coefficient (β; 95% CI). The significance was set < 0.05. Data were analyzed with SPSS version 23 (SPSS Inc., Chicago, IL, USA).

## Results

Accuracy of JL definition demonstrated a mean error of 0.3 ± 0.3 mm (range: 0 to 0.9 mm). ICC for fibular tip to JL distance was 0.96 (95% CI: 0.89-0.98).

**Fig. 4:**
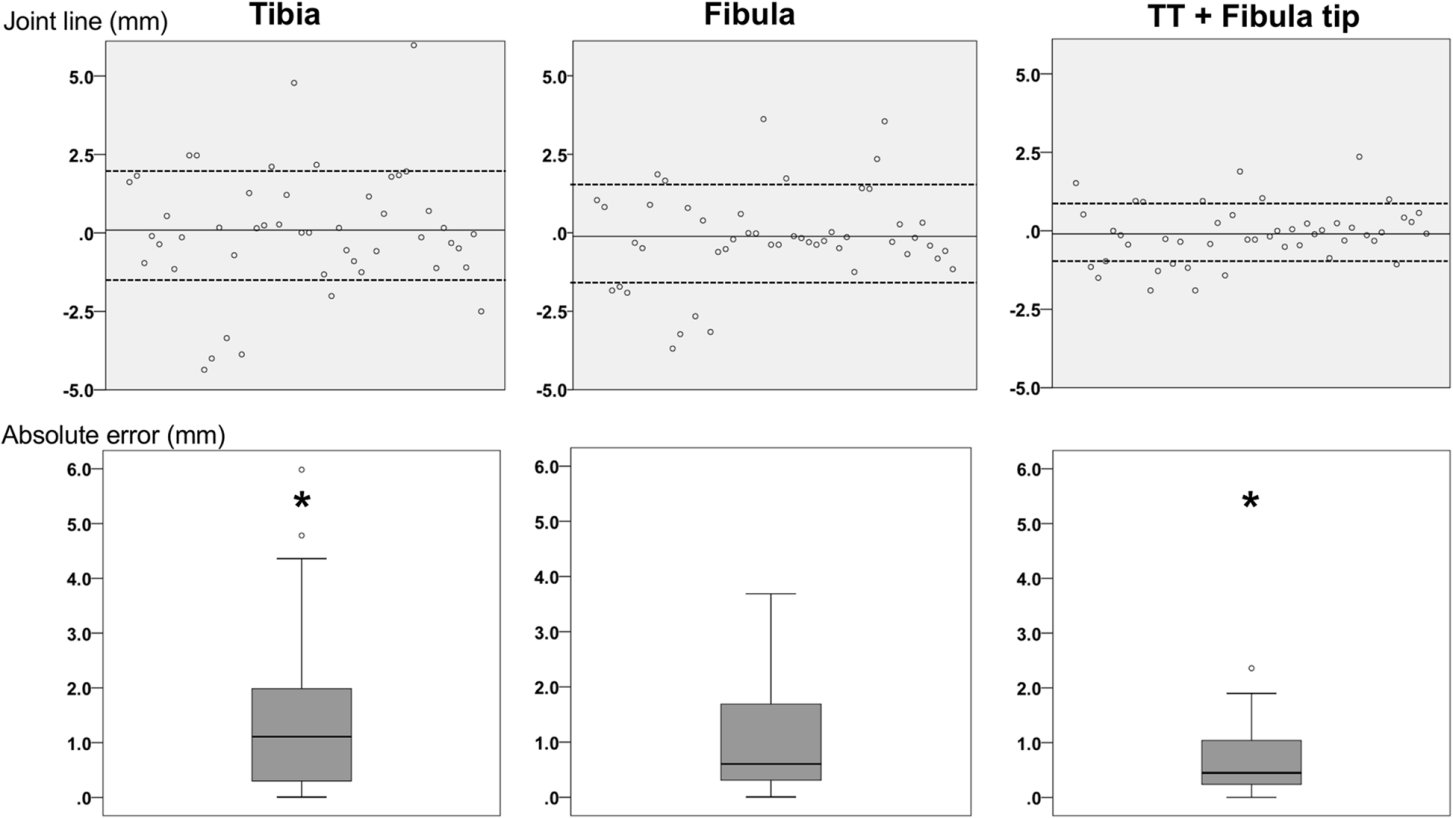
Mean joint line approximation and absolute error for each segment. TT: Tibial tuberosity. Top line: Y-axis depicts average JL approximation for each specimen and each segment in mm (bold: average, dotted: standard deviation). Bottom line: Y-axis depicts mean absolute JL error (line), IQR (box), range (whiskers) and outliers (points). Asterisks depict significant differences of mean absolute JL errors between segments (*p* = 0.03) after Bonferroni correction

Mean JL approximation did not yield significance among the three segments and was 0.1 ± 2.0 mm (range: − 4.4 to 6.0 mm) for the tibia, − 0.1 ± 1.5 mm (range: − 3.7 to 3.6 mm) for the fibula and − 0.1 ± 0.9 (range: − 1.9 to 2.4 mm) for TT and fibular tip (*p* = 0.76) (see Fig. 4).

JL error was highest for the tibia 1.4 ± 1.4 mm (range: 0 to 6.0 mm) and decreased for the fibula 0.8 ± 1.0 mm (range: 0 to 3.7 mm) and for TT and fibular tip segment 0.7 ± 0.6 (range: 0 to 2.4 mm) (*p* = 0.03) (see Fig. 4).

The linear regression model revealed a significant influence of tibia length side-to-side difference on JL error of tibia β:0.65 (*p* < 0.001) and fibula β:0.34 (*p* = 0.003). Fibular tip to JL distance side-to-side difference significantly influenced JL error of fibula β:0.37 (*p* = 0.03). No variables demonstrated a significant influence on JL error of TT and fibular tip (remaining regression coefficients listed in Table 1).

**Table 1:** Factors affecting mean absolute joint line error for each segment

	Counts (%) / mean ± SD	Tibia β (95% CI; ***p***-value)	Fibula β (95% CI; ***p***-value)	TT + Fibular tip β (95% CI; ***p***-value)
**Gender**				
• Male	34 (70.8%)^a^	0.23 (− 0.88-1.34; 0.68)	−0.25 (− 1.11-0.62; 0.57)	−0.11 (− 0.65-0.43; 0.69)
• Female	12 (25%)^a^			
**Height (cm)**	176.2 ± 8.6	−0.03 (− 0.08-0.03; 0.40)	0.03 (− 0.02-0.07; 0.21)	0.02 (− 0.01-0.04; 0.28)
**Weight (kg)**	83.1 ± 16.5	0.00 (− 0.01-0.01; 0.62)	0.00 (− 0.01-0.01; 0.58)	0.00 (− 0.01-0.01; 0.93)
**Tibia length (mm)**	2.1 ± 1.4^b^	0.65 (0.38-0.92; **< 0.001)**	0.34 (0.13-0.55; **0.003)**	0.12 (−0.02-0.25; 0.08)
**Fibula length (mm)**	2.9 ± 2.1^b^	0.06 (− 0.12-0.24; 0.49)	0.07 (− 0.07-0.20; 0.34)	0.07 (.-0.02-0.15; 0.12)
**Fibular tip to JL distance (mm)**	1.6 ± 1.1^b^	0.14 (− 0.28-0.56; 0.50)	0.37 (0.04-0.69; **0.029)**	0.16 (−0.04-0.36; 0.11)

Significant *p*-values marked bold

*β* Regression coefficient, *TT* Tibial tuberosity. JL: Joint line

^a^Two missing gender information

^b^Absolute side-to-side differences reported

Mean fibular tip to JL distance was 11.9 ± 3.4 mm (range: 3.4 to 22.1 mm) with a side-to-side difference of 1.6 ± 1.1 mm (range: 0 to 5.3 mm) and correlated significantly with height (*r* = 0.50; *p* < 0.001) but did not demonstrate significant gender differences (*p* = 0.78).

## Discussion

The most important finding of this study is that both the contralateral tibia and fibula can reliably be used to restore the original JL. The combined inclusion of TT and fibular tip for the registration protocol decreased JL error and approximated the JL more accurately.

The use of distinct anatomical landmarks for the contralateral 3D registration proved to reduce outliers and allows a more precise approximation of the original anatomy, which is in line with previous results [25]. The measured distance of the fibular tip to the JL yielded relatively small side-to-side differences and supports the role of the fibular tip as an important anatomical structure with an excellent inter-reader reliability, as previously demonstrated by Maderbacher et al. [14]. No influence of height, gender, and side-to-side differences on the JL error could be demonstrated for the TT and fibular tip segment, whereas the JL error of the tibia and fibula segment was dependent on side-to-side differences that have been analyzed in a previous study [17]. This represents a strength of the presented 3D registration method of TT and fibular tip compared to previous measurement methods based on absolute values and therefore being dependent on height [18] and require cumbersome ratios [16] or formula for conversion [13]. The accurate approximation of the JL is of high clinical relevance in the context of planning revision TKA when substantial bony defects occur. The fibula is rarely affected by bone loss and represents therefore a solid landmark in contrast to tibial and femoral based landmarks, even in TKA revision cases. As many surgeons start revision TKA with the tibia first, a tibia-based landmark is desirable. Overall, the use of the TT and fibular tip segment is preferable for 3D approximation of the JL, in our opinion. To apply the presented registration method a preoperative CT scan of the contralateral knee including the tibia and fibula segment of approximately ten centimeters distal to the joint line is required.

The accuracy to which extent the tibia JL needs to be restored remains debatable with contradicting results regarding its impact on functional outcome [2, 9, 24]. The surgical precision hardly allows to outperform the reported accuracy of the here presented JL error of 0.7 mm to date, even with the use of robotic systems [20]. Therefore, the reported accuracy is acceptable regarding the clinical relevance, in our point of view.

A kinematically well-balanced TKA requires an accurate joint line restoration according to the premorbid anatomy [12, 23]. These findings support the concept of restoring the original anatomy towards a personalized aligned TKA to improve functional outcome and mimic native tibiofemoral kinematic behavior [11]. To achieve this goal, the use of a 3D contralateral registration method to restore the individual anatomy would be of great help. Previously described drawbacks with the use of contralateral registration methods, as increased costs and radiation exposure are currently being tackled by adjusted CT protocols [21] and automatized segmentation protocols [19] and will likely continue to improve in the near future. Overall, the here presented results provide a reliable method to assess and restore the JL and might aid to improve patient outcome in TKA and revision TKA in the future.

The main limitation is that the presented method relies on a healthy contralateral anatomy for registration, at least in parts of the reported segments. Therefore, we analyzed different anatomical segments, to allow a registration, even in the presence of a partial deformity, a previous implanted contralateral TKA or degeneration of the contralateral side. Moreover, the osteoarthritis grade of the cadavers could not be assessed due the absence of complete knee radiographs. To address the limited availability of medical history of the cadavers, specimens with signs of deformities, previous surgeries or fractures were excluded.

## Conclusion

In conclusion, 3D registration of the contralateral tibia and fibula reliably approximated the original JL. The registration of, TT and fibular tip, as robust anatomical landmarks, improved the accuracy of JL restoration independent of tibia and fibula length side-to-side differences.

## Data Availability

The datasets used and/or analysed during the current study are available from the corresponding author on reasonable request.

## Abbreviations

3D: Three-dimensional
CT: Computer tomography
ICC: Intraclass correlation coefficient
ICP: Iterative closest point
JL: Joint line
OBB: Oriented bounding box
TKA: Total knee arthroplasty
TT: Tibial tuberosity
